# Analysis of the risk factors for severe lung injury after radical surgery for tetralogy of fallot

**DOI:** 10.3389/fsurg.2022.892562

**Published:** 2022-08-30

**Authors:** Yan He, Hong-Sheng Zhang, Ting-Zhou Zhang, Yu Feng, Yan Zhu, Xing Fan

**Affiliations:** Department of ICU in Pediatric Cardiology, Beijing An Zhen Hospital, Capital medical university, Beijing, China

**Keywords:** tetralogy of fallot, lung injury, pulmonary artery index, polymorphonuclear leukocyte, cardiopulmonary bypass

## Abstract

**Objective:**

This study aimed to determine the risk factors for severe lung injury (SLI) (partial pressure of oxygen/fraction of inspired oxygen <150) after radical surgery for tetralogy of Fallot with pulmonary stenosis (TOF/PS) in children.

**Method:**

A retrospective analysis was conducted including a total of 287 children with TOF/PS aged below 10 years (including 166 males) who had undergone radical surgery at the Center of Pediatric Heart Disease of the Beijing Anzhen Hospital (China) from 2018 to 2020.

**Results:**

A total of 83 cases (28.9%) had SLI after surgery. Univariate analysis showed that age, weight, pulmonary artery index (PAI), cardiopulmonary bypass (CPB) time, and polymorphonuclear leukocyte (PMN) percentage on the first day after surgery were risk factors for postoperative SLI. Multivariate logistic regression analysis showed that PAI, PMN percentage on the first day postoperatively, and CPB time were independent risk factors for SLI after surgery. The prediction model was established as follows: Logit(P) = 2.236 + 0.009*CPB-0.008*PAI-0.035*PMN, area under the curve (AUC) = 0.683, *P* < 0.001, sensitivity 65.8%, and specificity 68.6%. Following surgery, static lung compliance was significantly lower in the SLI group compared with the routine group. Complication rates and mortality were significantly higher in the SLI than in the routine group. Ventilator support times, the length of intensive care unit stays, and the total lengths of hospital stay were significantly longer in the SLI than in the routine group.

**Conclusion:**

The occurrence of SLI following radical surgery for TOF in children significantly affected postoperative recovery, and PAI, PMN percentage on the first day postoperatively, and CPB time were independent risk factors for SLI.

## Introduction

Congenital heart disease is one of the most common types of birth defects, among which tetralogy of Fallot (TOF) is the most common cyanotic congenital heart disease and has a high incidence in China where it accounts for 5%–7% of congenital heart diseases (1). Tetralogy of Fallot belongs to conus truncus malformation that includes four main manifestations, i.e., pulmonary artery stenosis, right ventricular hypertrophy, ventricular septal defect, and overriding aorta (2). Cardiopulmonary bypass (CPB) radical surgery can achieve satisfactory effects in terms of addressing these malformations. However, this type of surgery can cause ischemia-reperfusion injury and systemic inflammatory response, a synergistic effect that may result in the functional impairment of organs postoperatively (3, 4), particularly the lungs because of their high volume of inflammatory cells and dual-type blood supply.

A lung injury is more likely to occur after TOF surgery if abnormal development of the pulmonary vascular bed was present pre-surgery and due to an increase in pulmonary blood flow after radical surgery; therefore, a re-adaptation process must be implemented following TOF surgery.

Few recent reports exist on lung injuries following TOF surgery. The present paper presents a retrospective analysis of the risk factors for severe lung injury (SLI) following radical surgery for TOF/PS in children aged below 10 years. The research studied the differences in postoperative recovery between an SLI and routine group, respectively.

## Participants

A total of 287 children aged younger than/equal to 10 years who had received radical surgery for TOF/PS from 2018 to 2020 were included in this study. Those complicated with heart malformations other than atrial septal defects and patent ductus arteriosus were excluded. Pulmonary atresia was excluded.

## Methods

### Preoperative diagnosis

Some children evidenced no obvious symptoms; most of them, however, showed cyanosis, experienced feeding difficulty, squatting, and in severe cases, suffered from hypoxia. All patients were diagnosed by ultrasound, computed tomography angiography, or cardiac catheterization prior to surgery. If lateral branches sized ≤2 mm were present, no special treatment was conducted. Otherwise, right ventricular pulmonary angiography and aortic angiography were performed for the collateral occlusion of major aortopulmonary collateral arteries (MAPCAs).

The symptoms indicating the need for radical surgery included a pulmonary artery development index with a McGoon ratio >1.2 and a pulmonary artery index (Nakata index) >150 mm^2^/m^2^.

The following surgical methods were incorporated: (1) Right atrial incision or combined pulmonary artery incision for intracardiac correction was suitable for patients with a well-developed pulmonary valve annulus. (2) Standard widening of the right ventricular outflow tract (for which a Hegar probe with a bodyweight (kg) + 1 ∼ 2 mm could not be used). (3) For patients with severe dysplasia of the pulmonary valve annulus (Z < −3), right ventricular outflflow tract patch widening across the pulmonary valve annulus (TAP) remained the first choice. During the surgery, autologous pericardium or polytetrafluoroethylene (PTFE) sheets were used to create functional valves. Increasingly, surgeons are opting to use restrictive right ventricular small incisions (<0.5 cm) and right ventricular infundibulum sparing.

### Postoperative treatment

Continuous electrocardiogram monitoring and mechanical ventilation were routinely given to intensive care unit (ICU) patients following surgery. According to the postoperative circulation state, children were given cardiotonic and diuretic drugs as required (e.g., dopamine, 2–8 µg/kg.min; epinephrine, 0.02–0.1 µg/kg.min; milrinone, 0.1–0.4 µg/kg.min; furosemide, 0.1–0.4 mg/kg.h.

## Research methods

The following research indicators were included in the present study.

(1) Preoperative factors included gender, age, weight, preoperative transcutaneous oxygen saturation, hemoglobin, red cell distribution width, the incidence of an infection within three months preceding the surgery, pulmonary vascular McGoon Index, Nakata Index (PAI), and any MAPCA closure conducted prior to the surgery. Intraoperative factors were whether a second surgery had been performed, whether a transannular patch had been used, and CPB time. Postoperative factors included white blood cells (WBCs), polymorphonuclear leukocyte (PMN) percentage, and C-reactive protein (CRP) level on the first day after surgery, as well as a colloid infusion in the first three days after surgery.
(2) **Primary endpoint**. Severe postoperative lung injury: The minimum arterial partial pressure of oxygen/fraction of inspired oxygen (PaO_2_/FiO_2_, [P/F]) in the participants after surgery was retrospectively analyzed; thereafter, the participants were divided into two groups, i.e., the SLI group, which comprised patients with a P/F < 150 and the routine group, which comprised patients with a P/F ≥ 150.
(3) **Secondary endpoints**. The current study observed the following secondary endpoints: the incidence of death among the participants, other important complications (postoperative low cardiac output, severe arrhythmia, extracorporeal membrane oxygenation assistance, renal replacement therapy, severe central nervous system complications), mechanical ventilation time, minimum lung compliance (tidal volume/(peak inspiratory pressure -positive end- expiratory pressure)), and ICU and hospitalization stay times.
(4) The perioperative indices of the two groups were analyzed to determine the risk factors for SLI following radical TOF surgery. To explore the independent risk factors of SLI after a TOF/PS procedure, its cutoff value was derived and a prediction model was established accordingly.
(5) Differences in static lung compliance at the time point of minimum PaO_2_/FiO_2_ levels and the maximum vasoactive-inotropic score, complication rate, ventilator support time, length of ICU stay, and lengths of hospital stay after surgery were compared between the two groups.

### Statistical analysis

Normally distributed data were expressed as mean ± standard deviation and non-normally distributed data were expressed as quartiles. Risk factor analysis was conducted using univariate logistic regression, and significant indices (*P* < 0.05) were included in the multivariate regression analysis with conditional forward selection. For the correlation analysis of data between the two groups, an independent samples t-test was used for normally distributed data in the comparative study of postoperative recovery, and a nonparametric test was used for non-normally distributed data.

## Results

**Figure 1:**
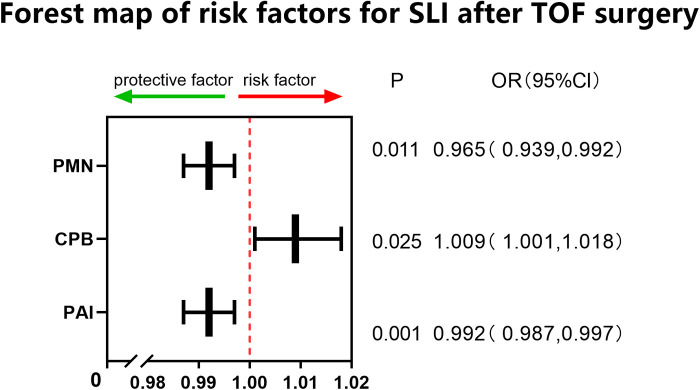
Correlation between Postoperative P/F and PAI. PAI, pulmonary artery index.

**Figure 2:**
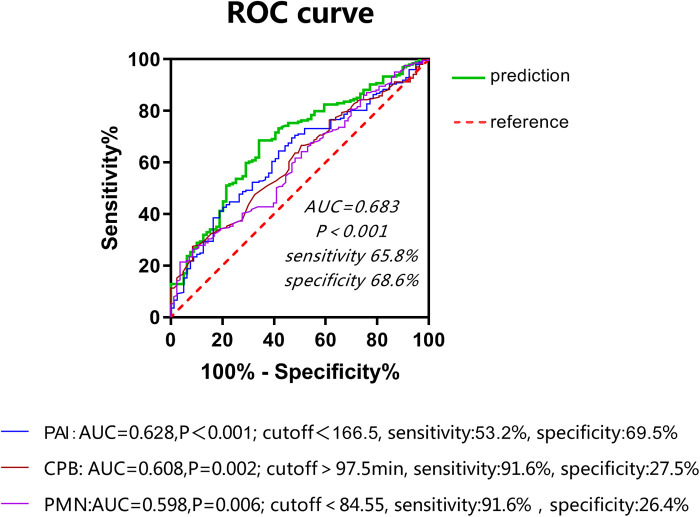
Correlation between Postoperative P/F and PMN Percentage. PMN, polymorphonuclear leukocyte.

This study involved a total of 287 children aged younger than or equal to 10 years (including 166 males) with an average age of 11 (8–23) months and weighing on average 9 (7.8–11) kg who received radical surgery for TOF/PS from 2018 to 2020. The overall minimum postoperative P/F was 202 (142,277.75); 83 (28.9%) cases were included in the SLI group. Univariate analysis showed that age, PAI, CPB time, and PMN percentage (all *P* < 0.01) on the first day after surgery were risk factors for SLI postoperatively. Multivariate logistic regression analysis showed that PAI (odds ratio [OR]: 0.992, 95% confidence interval [CI]: 0.987, 0.997, *P* < 0.01), PMN percentage on the first day after surgery (OR: 0.963, 95% CI: 0.937, 0.99, *P* < 0.01), and CPB time (OR: 1.009, 95% CI: 1.001, 1.018, *P* < 0.05) were independent risk factors for SLI after surgery (see Table 1 and Figure 1). The cutoff of PAI with SLI after TOF was less than 166.5 with a sensitivity of 53.2% and specificity of 69.5%. The cutoff time of CPB was >97.5 min, the sensitivity was 91.6%, and the specificity was 27.5%. The cutoff PMN was <84.55%, sensitivity was 91.6%, and specificity was 26.4%. The prediction model jointly established by the three was as follows: Logit(P) = 2.236 + 0.009*CPB-0.008*PAI-0.035*PMN, AUC = 0.683, *P* < 0.001. The sensitivity and specificity of this equation were 65.8% and 68.6%, respectively, which was better than a single prediction index (see Table 2 and Figure 2).

**Table 1:** Analysis of risk factors for SLI group and routine group after TOF surgery.

	Univariate analysis				Multivariate analysis			
		P/F < 150	P/F ≥ 150	*P*	OR	95% confidence interval (CI)		*P*
	*N* = 287	83(29%)	204(71%)					
Before surgery								
Gender (male)	166	49(59%)	117(57%)	0.796				
Age	11(8,23)	9.79(7.50,14.75)	11.70(8.51,27.55)	0.003				
Weight	9(7.8,11)	8.70(7.43,10.28)	9.01(7.92,12.35)	0.059				
SpO_2_	85.22 ± 7.66	85.04 ± 7.14	85.30 ± 7.88	0.793				
HB	138.57 ± 29.14	134.23 ± 28.09	140.33 ± 29.44	0.108				
RDW Red cell distribution width	40.39 5.33	40.35 ± 4.91	40.41 ± 5.51	0.93				
Any infection	29	9(11%)	20(10%)	0.827				
MCGOON	1.98 ± 0.59	1.90 0.76	2.01 0.51	0.142				
PAI	194.86 ± 65.23	174.40 52.18	203.07 68.19	0.000	0.992	0.987	0.997	0.002
Any MAPCA closure	24	9(11%)	15(7%)	0.351				
During surgery								
Whether second surgery was received	27	7(8%)	20(10%)	0.826				
Whether a transannular patch was used	116	40(48%)	76(37%)	0.058				
CPB time	120.13 36.18	129.31 41.24	116.40 33.30	0.006	1.009	1.001	1.018	0.025
Minimum temperature during CPB(°C)	28.45(27.42,29.76)	28.26(27.28,29.43)	28.62(27.5,29.91)					
After surgery								
P/F	202(142,277.75)	231(190.25,328)	121(99.18,135.9)	0.000				
WBCs on the first day	12.52 ± 3.98	12.83 4.38	12.39 3.81	0.401				
PMNs(%)	77.33 ± 9.99	74.77 9.56	78.38 9.99	0.005	0.963	0.937	0.990	0.008
CRP	25.07(16.07,40.49)	22.46(13.48,39.52)	25.76(16.98,41.87)	0.084				
Colloid infusion in the first three days	330(210,450)	350(221.25,461.67)	318(205.83,445)	0.304				

**Table 2:** Regression equationg for SLI after TOF surgery.

	B	Standard error	Wald	Significance	Exp (B)
PMN	−0.035	0.014	6.481	0.011	0.965(0.939,0.992)
CPB-min	0.009	0.004	5.051	0.025	1.009(1.001,1.018)
PAI	−0.008	0.003	10.113	0.001	0.992(0.987,0.997)
Constant	2.236	1.315	2.891	0.089	

**Figure 3:**
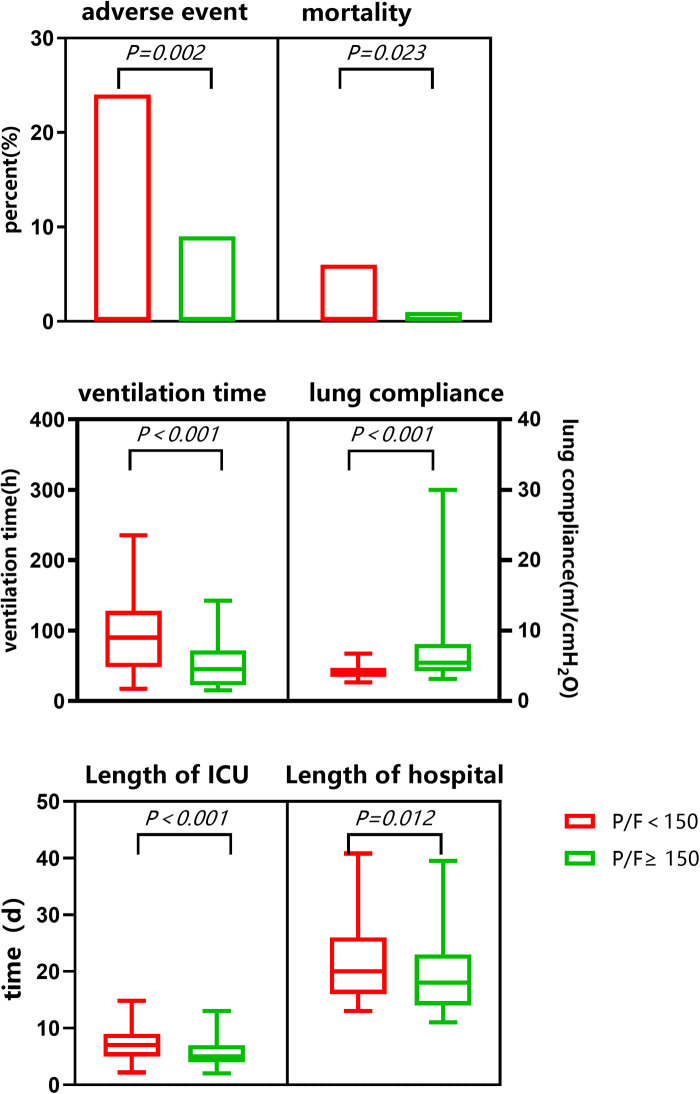
Correlation between Postoperative P/F and CPB Time. CPB, cardiopulmonary bypass.

There was a low correlation between the postoperative P/F and the PMN percentage on the first day after surgery (R^2^: 0.346, *P* 0.000) and little correlation between the postoperative P/F, PAI, and CPB time (see Figures 1–3).

Seven children died (mortality rate, 2.4%) and 39 other adverse events occurred (incidence rate, 13.6%). The average time of mechanical ventilation was 49 (24,93) h, the minimum postoperative lung compliance was 4.7 (4,6.7) ml/cmH_2_O, the time spent in ICU was 6 (4,8) d, and the hospitalization time was 19 (15,24) d *. Adverse event rates and mortality were significantly higher in the SLI than in the routine group. After surgery, static lung compliance was significantly lower in the SLI than in the routine group. Ventilator support time, length of ICU stay, and the total length of hospital stay were all significantly longer in the SLI than in the routine group (see Table 3 and Figure 3).

**Table 3:** Comparison of recovery between SLI group and routine group after TOF surgery.

	Lung compliance (ml/cmH_2_O)	Adverse event rate	Mortality	Ventilator support time (h)	Length of ICU stay (d)	Length of hospital stay (d)
	4.7(4,6.7)	39	7	49(24,93)	6(4,8)	19(15,24)
P/F < 150	4.08(3.43,4.8)	20(24%)	5(6%)	90(47.83,127.4)	7.14(5.12,9.46)	20.38(16.08,26.1)
P/F ≥ 150	5.39(4.17,7.98)	19(9%)	2(1%)	45.02(22.42,71.67)	5.33(3.52,7.39)	18.23(14.24,22.93)
*P*	0.000	0.002	0.023	0.000	0.000	0.012

## Discussion

The degree of pulmonary vascular development in TOF is an important factor for prognosis determination and in dictating delayed extubation postoperatively (5). In China, TOF radical surgery is generally performed at an older age (average age, 11 months). At this age, pulmonary vascular bed development has been affected. Due to the presence of pulmonary artery stenosis in TOF, pulmonary artery blood flow will increase following radical surgery. In some children, pulmonary blood flow distribution becomes uneven after surgery due to chronic hypoxia and the formation of MAPCAs. As a result, the pulmonary vascular bed requires re-adaptation, and lung injury can easily occur postoperatively. Acute lung injury (ALI) is defined as a P/F  < 300 that results due to a cause other than left ventricular dysfunction. The mean P/F of 202 (142,−277.75) among the children showed that although various lung protection strategies had been adopted in the perioperative period, the incidence of lung injury remained high. In this study, participants with a P/F < 150 were included in the SLI group (incidence, 28.9%). The diagnostic criteria (draft) for Acute lung injury/acute respiratory distress syndrome (ALI/ARDS) formulated by Chinese Respiratory Society in 2000 are as follows: PaO_2_/FiO_2_ ≤ 40 kPa(300 mmHg, 1 mmHg = 0.133 kPa) is ALI, PaO_2_/FiO_2_ ≤ 27 kPa(200 mmHg) is ARDS. Berlin Standard 2012: 200 mmHg < PaO_2_/FiO_2_ ≤ 300 mmHg is mild ARDS; 100 mmHg < PaO_2_/FiO_2_ ≤ 200 mmHg is moderate ARDS; PaO_2_/FiO_2_ ≤ 100 mmHg is severe ARDS (6). This study combines the above two standards with 150 mmHg as the cut-off point. The authors hypothesized that said degree would also impact the occurrence of lung injury postoperatively. Therefore, the pulmonary vascular McGoon Index and PAI were included in this research. These two indices differ in that the McGoon Index focuses on the aortic diameter of children and is used for correcting pulmonary vascular development, while the PAI is used for correcting differences in children of different ages using the body surface area thereof.

The PAI significantly impacted the occurrence of SLI after surgery and was an independent risk factor for the occurrence of such an injury. The cutoff value was 166.5, which indicated that the PAI was less than 166.5; this gave rise to a higher risk of postoperative SLI and indicated that the impact of the degree of pulmonary vascular development on prognosis in cases of TOF may be reflected by the presence of SLI postoperatively. These results indicated that the McGoon Index provided no statistical significance. Existing studies have also indicated PAI to be more sensitive to pulmonary vascular development compared to using the McGoon Index and indicating a better correlation with postoperative recovery (7). This may be related to the decrease of left cardiac circulation after pulmonary vascular dysplasia, which also to some degree affects the inner diameter of the aorta. For patients with PAI < 166.5, the ICU team should be alert to the risk of postoperative SLI. Lung injury is a common complication following CPB surgery for congenital heart disease. The CPB process can cause ischemia-reperfusion injury and systemic inflammatory responses, a synergistic effect that may impact pulmonary vessels and lung parenchyma. The impact on pulmonary vessels manifests as increased permeability, interstitial edema, microvasoconstriction, and increased resistance. The impact on alveoli manifests as fluid accumulation inside the alveoli and reduced surface-active substances, lung compliance, and gas exchange function due to an impaired vascular barrier. Ischemia-reperfusion injury and systemic inflammatory response also impact and exacerbate one another; their primary clinical manifestations include hypoxemia, tachypnea, and diffuse infiltrative shadows in both lungs according to chest radiography; in some patients, complications such as pulmonary atelectasis and pneumothorax can occur (8).

Almost all patients experience different degrees of pulmonary hypofunction after undergoing CPB surgery, ranging from subclinical functional changes in mild cases to ALI or even acute respiratory distress syndrome in severe cases with a mortality rate as high as 46% (9–11).

Existing studies have shown that CPB time and intraoperative minimum temperature may affect ventilator support time postoperatively (12). This study included these two variables, and the results showed that CPB time significantly impacted the occurrence of lung injury after TOF surgery and was thus an independent risk factor for said injury. The cutoff value was 97.5 min, which indicated that the risk of postoperative SLI was higher in the group of children with a CPB time >97.5 min. Therefore, reducing CPB time is an important measure for reducing lung injury after surgery.

The intraoperative minimum temperature had no statistical significance in this study and, unlike existing studies, the intraoperative minimum temperature was lower in the SLI group than in the routine group. A possible reason for this may have been the longer CPB time in the SLI group and, accordingly, more active cooling measures were taken to protect the functioning of organs.

This study highlighted the PMN percentage on the first day after surgery as an independent risk factor for SLI; this value was 74.77% ± 9.56% in the SLI group, which was significantly lower than the 78.38% ± 9.99% result in the routine group. Additionally, WBCs and CRP values were both higher than normal after surgery but did not differ significantly between the two groups. Existing studies have shown that PMNs are an important component in ischemia-reperfusion injury and systemic inflammatory response during the CPB process as well as in lung injury. The systemic inflammatory response first activates the complement system and chemotactically activates PMNs, which interact with pulmonary vascular endothelial cells to release inflammatory mediators that trigger an inflammatory cascade as well as a sustained release of toxic substances that cause lung injury (13–15). Ischemia-reperfusion injury causes disequilibrium between superoxide dismutases and peroxidases, which leads to oxidative stress and the activation of PMNs and other inflammatory cells that release, among others, large volumes of elastases that destroy lung tissue (16–18). According to existing studies, WBCs, PMN, CRP, and PCT are all involved in systemic inflammatory responses following CPB and are sometimes related to nosocomial infection after congenital heart disease surgery (19, 20). In recent years, the use of a leukocyte filter to improve postoperative lung function during CPB has also received extensive attention (21–23).

The present study showed that the PMN percentage was lower in the SLI group than in the routine group; a possible reason for this was that the PMN compensatory increase ability of the SLI group had been insufficient and more PMNs were chemotactically activated in the lungs to participate in the process of lung injury. As a result, the PMN percentage was lower in this group than in the routine group and became an independent risk factor for SLI. Its cutoff value was 84.55%, which indicated that the risk of postoperative SLI was higher in this group of children where the PMN value was <84.55%. Because the lowest postoperative P/F value of many children occurred on the second or even the third day after surgery. Therefore, the ICU team is advised to be alert to the risk of SLI for children with PMN < 84.55% on the first day after surgery, avoid excessive active disengagement from the ventilator, and reduce the risk of secondary intubation.

Existing studies showed that children with SLI after surgery had longer ventilator support times and lengths of ICU stay, increased related complications, and reduced postoperative survival, which aggravated the suffering of families and the burden on society (24). In this study, compared to the routine group, the SLI group had reduced static lung compliance with ventilator support in addition to significant hypoxia. The mortality rate was as high as 6% in the SLI group, 2.5 times the overall mortality in the cohort, and six times that in the routine group. Furthermore, the rates of other adverse events were significantly higher in the SLI than in the routine group. Ventilator support time, length of ICU stay, and length of hospital stay were all significantly longer in the SLI group.

The above results indicated that the complication and mortality rates were significantly higher and recovery was delayed in those with SLI after radical treatment for TOF. A patient's death and serious adverse events could also occur synchronously with SLI. There is not necessarily a causal relationship between them and, accordingly, the mechanical ventilation time will be delayed. Increased ICU and hospitalization stays may also be the result of other adverse events that are not necessarily caused entirely by SLI.

In this study, the age at surgery was a significant factor in the univariate analysis; the lower the age, the more likely the patient was to develop SLI following surgery. However, the multivariate analysis showed that age was not an independent risk factor for SLI. Young age was also a high-risk factor for developing a systemic inflammatory response after surgery. Pulmonary vascular development was also important for SLI after TOF surgery and older age at surgery indicated poorer development of the pulmonary vascular bed. This may have partially countered the impact of age on SLI after surgery.

The surgery method and the presence/absence of systemic pulmonary collateral will also have an impact on postoperative recovery. This study included two variables, i.e., whether or not to cross-ring patch and whether or not to block collateral prior to conducting surgery but no significant results were obtained. It is possible that the indications of cross-ring patch and large collateral occlusion had been accurately selected in this study and that the surgery techniques had been properly applied, thereby circumventing an impact on postoperative SLI.

The present study showed that SLI following surgery for TOF in children may have affected their recovery and increased their complications and mortality. Furthermore, PAI, PMN percentage on the first day after surgery, and CPB time were identified as independent risk factors for SLI. Among these, CPB time and postoperative PMN had high overall sensitivity but poor specificity; this indicated that false-positive results could easily occur when SLI was predicted only by these two variables. Conversely, PAI sensitivity and specificity were not very high. The prediction accuracy using only this variable was thus not satisfactory.

The established prediction model, based on the above results, had an AUC of 68.3% and both the sensitivity and specificity values were good; accordingly, the prediction model should be used to jointly predict. In addition, this study adopted a single-center retrospective analysis and only studied the data on the first day after surgery, as PMNs, WBCs, and CRP tend to change over time postoperatively. The impact of changes in inflammatory cells on lung injury should be further observed in future studies.

## Conclusion

This retrospective cohort study showed that patients complicated with SLI after radical surgery for TOF required longer ventilator support times and experienced increased complication and mortality rates. Pulmonary artery index, PMN percentage on the first day after surgery, and CPB time were independent risk factors for SLI. This study adopted a single-center retrospective analysis and only studied the data on the first day after surgery because PMNs, WBCs, and CRP tend to change over time postoperatively. In future studies, the change tendency among inflammatory cells should be further observed and bronchoalveolar lavage inflammatory markers should also be studied to gain a more thorough and complete understanding of lung injury after CPB surgery.

## Data Availability

The original contributions presented in the study are included in the article/Supplementary Material, further inquiries can be directed to the corresponding author/s.
